# Prevalence, antimicrobial susceptibility profile and predictors of asymptomatic bacteriuria among pregnant women in Adigrat General Hospital, Northern Ethiopia

**DOI:** 10.1186/s13104-018-3844-1

**Published:** 2018-10-19

**Authors:** Senait Tadesse, Tsega Kahsay, Gebre Adhanom, Getachew Kahsu, Habtom Legese, Aderajew G/wahid, Awoke Derbie

**Affiliations:** 1 College of Medicine and Health Sciences, Adigrat University, Adigrat, Ethiopia; 2 Department of Medical Microbiology, Immunology and Parasitology, College of Medicine and Health Sciences, Bahir Dar University, Bahir Dar, Ethiopia; 3 The Centre for Innovative Drug Development and Therapeutic Trials for Africa (CDT-Africa), Addis Ababa University, Addis Ababa, Ethiopia

**Keywords:** Asymptomatic bacteriuria, Antimicrobial susceptibility profile, Pregnant women, Adigrat General Hospital

## Abstract

**Objective:**

Approach to asymptomatic bacteriuria among pregnant women in Ethiopia is mainly based on clinical grounds and urine strip and microscopy tests. On top of this, the treatment is also on an empirical basis which may leads to an increased antimicrobial resistance. The aim of this study was to assess the prevalence, antimicrobial susceptibility profile and associated factors of asymptomatic bacteriuria among pregnant women attending antenatal clinic in Adigrat Hospital, Northern Ethiopia.

**Results:**

Out of 259 pregnant women included in the study, the prevalence of asymptomatic bacteriuria was at 55 (21.2%). Gram negative bacteria, specifically *Escherichia coli* were the predominant isolates followed by *Klebsiella* species and *Proteus mirabilis.* Of the Gram positive identified bacteria*, Staphylococcus aureus* was main isolate. Age of the mother (18–25 years old) with [AOR = 8.5, 95% CI (2.2, 32.9)], family income (< 1000 ETB) with [AOR = 7.5, 95% CI = (2.4, 23.1)] and gestational period at 1st trimester [AOR = 11.9, 95% CI (4.4, 32.4)] and 2nd trimester [AOR; 5.6, 95% CI (2.0, 15.5%)] were predictors significantly associated with asymptomatic bacteriuria. All Gram negative isolates were found 100% resistance to Ampicllin. Moreover, all Gram positive isolates were found sensitive to Vancomycin at 100%.

## Introduction

Urinary tract infection (UTI) is caused by the growth of pathogens anywhere in the urinary tract [1]. Bacteria which reside in the digestive tract, vagina or around the urethra remain the major agents of UTI [2]. *Escherichia coli* are the most frequent organisms isolated from subjects with UTI and are responsible for 75–80% of the cases [3–5].

Urinary tract infection affects all age groups, but women particularly pregnant women are more susceptible than men, due to thier short urethra and easy contamination of urinary tract with fecal flora [6]. In addition, lower socioeconomic status, history of catheterization, multiparity and past history of urinary tract infection may contribute to the development of UTI in pregnancy [7–11]. Pregnancy by itself increases the risk of UTIs due to hormonal changes and expanding uterus that put pressure on the bladder leads slow urine output which results favorable growth condition for bacteria and increase UTI in pregnancy. But in many cases the infection is asymptomatic [12].

Asymptomatic bacteriuria (ABU) is the presence of a significant level of bacteria in the urine in the absence of clinical signs or symptoms of urinary tract infection [13, 14]. Asymptomatic bacteriuria is one of the major risk factors for the development of UTIs during pregnancy which accounts for about 70% of the cases [15]. If untreated, it causes about 40% cystitis and 30% pyelonephritis which might lead to delivery of premature or low-birth-weight infants [3], intrauterine growth retardation, preterm labor, intrauterine fetal death, and increased prenatal mortality and morbidity [16].

The incidence of maternal complications such as anemia, preeclampsia, renal failure, and septicemia [17] can be decreased by early screening and treating promptly of ABU during pregnancy [18–20]. According to the American College of Obstetricians and Gynecologists, screening of ABU is recommended in all pregnant women [21, 22]. Pregnant women with ASB should receive antibiotic therapy directed at the cultured organism [23]. So, screening for ASB has been included as one of the cost-effective strategies for improving maternal and neonatal health [24]. Nevertheless, unreliable tests are used to detect ABU [25, 26] and antimicrobials are widely used empirically, especially in the developing world, like Ethiopia [27]. The impact of antimicrobial overuse on the antimicrobial susceptibility of human pathogens impairs the effectiveness of current and future antimicrobial agents and emergence of resistant bacterial infections has been increasing [28].

Currently, in Ethiopia data regarding the prevalence of ASB and antibiotic susceptibility patterns among pregnant women have been released at different health facility [28, 29]. However, such data are missing from the study area and the antibiotic susceptibility patterns vary according to regional and geographical location and also change through time. Therefore, this study was aimed at assessing the prevalence, antimicrobial susceptibility profile and associated factors of ASB among pregnant women in Adigrat General Hospital, Northern Ethiopia.

## Main text

### Methods

A cross-sectional study was conducted at Adigrat General Hospital, Northern Ethiopia from 1 January to 30 April 2018. All pregnant women without sign and symptoms of UTI and who did not take antibiotics in the last 2 weeks at the time of data collection were included in the study. The sample size was calculated using single population proportion formula at 18.8% prevalence [29] and 95% confidence level, the total sample size was at 259.

Demographic related data were collected using structured questionnaire. About 5 ml freshly voided midstream urine sample was collected using sterile plastic containers for bacteriological culture and antimicrobial susceptibility testing. Colony counts yielding bacterial growth of ≥ 10^5^ CFU/ml of urine were considered as significant bacteriuria. Bacterial isolates were identified as per the standard bacteriological procedure using colony characteristics, gram-staining, and series of biochemical tests.

Antimicrobial susceptibility testing was performed using disk diffusion method on Mueller–Hinton agar medium (Oxoid Basingstoke, UK) based on  the Clinical and Laboratory Standards Institute (CLSI) guide lines [30].

Data were  entered and analyzed using SPSS version 20 for Windows. Stepwise logistic regression analysis was considered to determine factors associated with ASB. Odds ratio (OR) and 95% confidence interval (CIs) were calculated to measure the strength of the association. *P* value of < 0.05 was considered as statistically significant.

### Results

#### Socio-demographic characteristics of the participants

A total of 259 pregnant women were included in this study. The mean age of the study participants was at 26.3 years with standard deviations (SD) of 5.4 (ranged 18–25 years). Majority of participants were married (93.1%), house wife (73.0%), urban dwellers (90.7%) and attended high school (48.6%). Similarly, more than half of the study subjects at (56.4%) were multigravida and with regard to their gestation period, about (47.5%) of the subjects were at 3rd trimester (47.5%) and (27.4%) were at 2nd trimester. On top of these, (15.5%) and (8.1%) of the study participants had history of diabetes and UTI, respectively. About 8.5% of the participants were HIV positive.

#### Prevalence of asymptomatic bacteriuria and types of bacterial isolates

From the total analyzed urine samples, 55 (21.2%) were found positive for significant bacteriuria (CFU ≥ 10^5^/ml). Of these, seven different bacterial isolates were identified of which  the majority of the isolates at 38 (64.1%) were Gram negative bacteria. *E. coli* was found the most frequent isolate at 19 (34.6%), followed by *S. aureus* (18.2%), *Klebsiella* species (18.2%), *S. saprophyticus* (12.7%), *P. mirabilis* (9.1%) and *Enterobacter* species (1.8%).

#### Factors associated with asymptomatic bacteriuria

All bivariate results that had a *P* value of less than 0.2 were subjected to multivariate logistic regression analysis to controls the undesirable effects of confounding variables and we found that age of the mothers (18–25 years old) with [AOR = 8.5, 95% CI (2.2, 32.9), *P* value = 0.001] family income level (< 1000 ETB) with] AOR = 7.5, 95% CI = (2.4, 23.1), *P* value = 0.001] and gestational period (1st trimester with, [AOR = 11.9, 95% CI (4.4, 32.4), *P* value = 0.001] and (2nd trimester) with, [AOR; 5.6, 95% CI (2.0, 15.5%), *P* value = 0.01] were found significantly associated with asymptomatic bacteriuria (Table 1).

**Table 1:** Bivariate and multivariate analysis of factors associated with asymptomatic bacteriuria among pregnant women attending at Adigrat general hospital, Northern Ethiopia, 2018

Variables	Categories	ASB		COR (95% CI)	AOR (95% CI)	*P* value
		Yes	No			
Age	18–25	40	87	3.6 (1.3, 9.7)	8.5 (2.2, 32.9)	0.001
	26–33	10	78	1.3 (0.3, 3.2	1.6 (0.4, 6.6)	0.5
	34–45	5	39	1	1	
Gestational period	1st trimester	25	40	5.8 (2.6, 12.6)	11.9 (4.4, 32.4)	< 0.001
	2nd trimester	18	53	3.1 (1.4, 6.9)	5.6 (2.0, 15.5)	0.01
	3rd trimester	12	111	1	1	
Income	> 1000	22	18	7 (2.8, 18.5)	7.5 (2.4, 23.1)	0.001
	1000–1999	13	56	1.4 (0.5, 3.4)	0.96 (0.3, 2.7)	0.9
	2000–2999	11	77	0.8 (0.3, 2.2)	0.6 (0.2, 1.8)	0.3
	> 2999	9	53	1	1	

#### Antimicrobial susceptibility profile of the isolates

The detail antimicrobial susceptibility profile of the isolates is presented in (Table 2). In this study, Gram negative isolates were found sensitive to CRO at (86.8%), CIP (77.1%), AMC (76.3%) and CN (76.3%). On the other hand all Gram negative isolates were found fully resistance (100%) to AMP. Gram-positive isolates were relatively found sensitive to VAN at (100%), CRO (88.2%), CIP (82.3%) and F (76.5%) and in contrast found resistance to SXT at (64.7%).

**Table 2:** Antimicrobial susceptibility pattern of bacterial isolates from asymptomatic bacteriuria among pregnant women attending at Adigrat hospital, Nortern Ethiopia, 2018

Type of isolates	Antimicrobial resistance profile of the isolates, frequency (%)								
G−ve isolates	Profile	CN	AMC	Amp	CIP	SXT	CRO	F	
*E. coli* (19)	S	15 (78.9%)	16 (84.2%)	0 (0%)	14 (73.6%)	9 (47.4%)	17 (89.4%)	14 (73.6%)	
	I	0 (0%)	1 (5.2%)	0 (0%)	1 (5.2%)	1 (5.2%)	1 (5.2%)	0 (0%)	
	R	4 (21.1%)	2 (10.5%)	19 (100%)	4 (21.5%)	9 (47.4%)	1 (5.2%)	5 (26.6%)	
*Klebsiella* species (10)	S	7 (70%)	8 (80%)	0 (0%)	7 (70%)	6 (60%)	9 (90%)	6 (60%)	
	I	0 (0%)	1 (10%)	0 (0%)	0 (0%)	0 (0%)	0 (0%)	0 (0%)	
	R	3 (30%)	1 (10%)	10 (100%)	3 (30%)	3 (30%)	1 (10%)	4 (40%)	
*P. mirabilis* (5)	S	4 (80%)	3 (60%)	0 (0%)	4 (80%)	3 (60%)	4 (80%)	4 (80%)	
	I	0 (0%)	0 (0%)	0 (0%)	0 (0%)	0 (0%)	0 (0%)	0 (0%)	
	R	1 (20%)		10 (100%)	1 (20%)	2 (40%)	1 (20%)	1 (20%)	
*P. aeruginosa* (3)	S	2 (66.7%)	1 (33.3%)	0 (0%)	1 (33.3%)	0 (0%)	2 (66.7%)	2 (66.7%)	
	I	0 (0%)	0 (0%)	0 (0%)	0 (0%)	0 (0%)	0 (0%)	0 (0%)	
	R	1 (33.3%)	2 (66.7%)	3 (100%)	2 (66.7%)	3 (100%)	1 (33.3%)	1 (33.3%)	
*C. freundii* (1)	S	1 (100%)	1 (100%)	0 (0%)	1 (100%)	0 (0%)	1 (100%)	0 (0%)	
	I	0 (0%)	0 (0%)	0 (0%)	0 (0%)	0 (0%)	0 (0%)	0 (0%)	
	R	0 (0%)	0 (0%)	1 (100%)	0 (0%)	1 (100%)	0 (0%)	1 (100%)	
Total (38)	S	29 (76.3%)	29 (76.3%)	0 (0%)	29 (77.1%)	18 (47.4%)	33 (86.8%)	26 (68.4%)	
	I	0 (0%)	2 (5.2%)	0 (0%)	1 (2.6%)	1 (2.6%)	1 (2.6%)	0 (0%)	
	R	9 (23.7%)	7 (18.4%)	38 (100%)	8 (21.1%)	19 (50%)	5 (13.2%)	12 (31.6%)	

Type of isolates	Antimicrobial resistance profile of the isolates, frequency (%)								
G+ isolates	Profile	Pen	Ery	CL	CIP	SXT	CRO	F	VAN
*S. aureus* (10)	S	7 (70%)	6 (60%)	6 (60%)	8 (80%)	4 (40%)	9 (90%)	6 (60%)	10 (100%)
	I	0 (0%)	0 (0%)	0 (0%)	1 (10%)	0 (0%)	0 (0%)	0 (0%)	0 (0%)
	R	3 (30%)	4 (40%)	4 (40%)	1 (10%)	6 (60%)	1 (10%)	4 (40%)	0 (0%)
*S. saprophyticus* (7)	S	5 (71.4%)	6 (85.7%)	6 (85.7%)	6 (85.7%)	2 (28.5%)	6 (85.7%)	6 (85.7%)	7 (100%)
	I	0 (0%)	0 (0%)	1 (14.3%)	0 (0%)	0 (0%)	0 (0%)	0 (0%)	0 (0%)
	R	2 (28.6%)	1 (14.3%)	0 (0%)	1 (14.3%)	5 (71.5%)	1 (14.3%)	1 (14.3%)	0 (0%)
Total (17)	S	12 (70.5%)	12 (70.5%)	12 (70.5%)	14 (82.3%)	6 (35.3%)	15 (88.2%)	13 (76.5%)	17 (100%)
	I	0 (0%)	1 (5.9%)	1 (5.9%)	1 (5.9%)	0 (0%)	0 (0%)	0 (0%)	0 (0%)
	R	5 (29.5%)	4 (23.5%)	4 (23.5%)	2 (11.8%)	11 (64.7%)	3 (17.7%)	4 (23.5%)	0 (0%)

*CN* gentamicin, *AMC* amoxicillin clavulanic acid, *AMP* ampicillin, *CIP* ciprofloxacin, *SXT* trimethoprim/sulfamethoxazole, *CRO* ceftriaxone, *F* nitrofurantoin, *P* penicillin, *Ery* erythromycin, *CL* clindamycin, *VAN* vancomycin, *S* sensitive, *I* intermediate, *R* resistant

### Discussion

In Ethiopia, government owned health facilities provide antenatal care services for free. However, there is no routine urine culture test for pregnant women to screen for ASB during the follow up period, instead they get tested for urinalysis using urine strip tests and treatment is based on the empirical basis. Early detection of ASB during pregnancy has paramount importance to prevent its bad consequences [31]. It is good to know the level of ASB and current antimicrobial resistance profile of the isolates for service auditing.

The prevalence of ASB among pregnant women in the present study was at 21.2%. We noticed that the prevalence rate of ASB is varied in different studies. Our finding was found higher than other studies conducted in different parts of Ethiopia (10.6–11.5%) [27, 32, 33], and elsewhere in other African countries at (7.3–14.7%) [3, 4, 34–36], Chandanaish, Bangladesh at (13%) [5], and Sanandaj, Iran at (8.9%) [7]. However, our report was found almost comparable with other reports in Ethiopia that reported ASB between 16% and 18.9% [29, 37, 38] and abroad in Sagamu, Nigeria (23.9%) [39]. On the other hand, our finding was lower than reports from a study done in Nigeria (26.7–45.3%) [40, 41] and Iran (29.1%) [26]. The wide-ranging reported prevalence of ASB across different studies from one country to other and among region of the same country might be attributed to the difference in associated factors, sample size, geographical variations, and social habit of the community and health education practice.

In this study, Gram-negative bacterial isolates specifically *E. coli* (34.6%), *Klebsiella* species (18.2%), *P. mirabilis* (9.1%) and *P. aeruginosa (5.5%)* were more prevalent than their counterparts; *S. aureus* (18.2%), *S. saprophyticus* (12.7%) and *Enterobacter* species (1.8%). Similar findings were also reported in Ethiopia (Dire Dawa and Adama) and in abroad, (Nigeria) [27, 37, 40]. This is in line with the fact that most uropathogenic bacteria are Gram negatives that usually sourced from the bowel and ascend to the urinary tract. They have also a unique structure (pilus adhesions) which help the bacteria for attachment to the uroepithelium lining  and prevent them from urinary lavage, allowing for multiplication and tissue invasion resulting in invasive infections in pregnancy [42].

With regard to factors associated with ASB, we revealed that women’s age, income and gestational period were found significantly associated with ASB (*P* value < 0.05). Pregnant women in the age groups of 18–25 years old were found more than eight times [AOR = 8.5, 95% CI (2.2, 32.9)] more likely to positive for ASB than the old age groups. This may be explained by the fact that early and intensive sexual intercourse which may cause minor urethral trauma and transfer bacteria from the perineum into the bladder. Similar finding was also reported in the previous studies [27, 40, 43, 44]. Similarly, pregnant women with low family income [AOR = 7.5, 95% CI = (2.4, 23.1)] were found seven times more likely to positive for ASB. This could be due to the relation of low socio economic status with nutrition and immunity especially in pregnant women. Similar finding was reported in another a study done in other parts of Ethiopia [38].

Finally, in this study we reported that pregnant women at their 1st [AOR = 11.9, 95% CI (4.4, 32.4)] and 2nd [AOR; 5.6, 95% CI (2.0, 15, 5%)] trimester gestational period were also found more likely to be positive for ASB that women at the 3rd trimester. This might be due to the fact that ASB/UTI in pregnant women usually begins within week 6 and peaks during 22–24 of pregnancy due to urethral dilation, increased bladder volume and decreased bladder tone along with decreased urethral tone, which encourage bacterial growth in the urine. It is also indicated that the gold standard for screening ASB is in early pregnancy of 12–16 weeks [45]. Similar finding was also reported in another study [36].

Concerning antimicrobial resistance profile of the isolates, in the present study Gram negative isolates were found with higher rate of resistance to the commonly prescribed antibiotics in the country. All Gram negative isolates were resistance to Ampicillin (100%). Other studies done in Ethiopia and abroad have also reported comparable finding on this regard [3, 27, 46, 47]. Most of the commonly prescribed  antimicrobials are freely available in local pharmacies and people could purchase and use them without prescription in Ethiopia which might also contribute its share for drug resistance. On the other hand, in this study Gram positive bacterial isolates were found relatively sensitive to VAN (100%), CIP (82%), and CRO (88%) in which these reports are in agreement with the finding of other study [27].

### Conclusions

The prevalence of ASB among pregnant women in the present study was at 21.2%. Women’s age, income and gestational period were found significantly associated with ASB. The most frequent identified isolates were *E. coli* (34.6%), followed by *Klebsiella* species (18.2%) and *S. aureus* (18.2%). All Gram negative isolates were resistance to Ampicillin at (100%). Therefore, screening and treatment of pregnant women for ASB, specifically at their 1st and 2nd trimester, should be considered in antenatal care practices and there is a need for periodic surveillance of the type of bacterial pathogens and their updated antimicrobial resistance profile in the study setting.

## Limitations

In this study authors collected urine sample once regardless of the participants’ period of gestation, this could potentially influence to disclose the actual status of ASB during the entire period of pregnancy. Our study also lacks data on extended spectrum beta lactamase production status of some the isolates due to resource constraints.

## Abbreviations

ASB: asymptomatic bacteriuria
CFU: colony forming unit
DM: diabetic mellitus
HIV: human immunodeficiency virus
UTI: urinary tract infection
