# Correction to: Prevalence, antimicrobial susceptibility profile and predictors of asymptomatic bacteriuria among pregnant women in Adigrat General Hospital, Northern Ethiopia

**DOI:** 10.1186/s13104-018-3911-7

**Published:** 2018-11-08

**Authors:** Senait Tadesse, Tsega Kahsay, Gebre Adhanom, Getachew Kahsu, Haftom Legese, Aderajew G/wahid, Awoke Derbie

**Affiliations:** 1 College of Medicine and Health Sciences, Adigrat University, Adigrat, Ethiopia; 2 Department of Medical Microbiology, Immunology and Parasitology, College of Medicine and Health Sciences, Bahir Dar University, Bahir Dar, Ethiopia; 3 The Centre for Innovative Drug Development and Therapeutic Trials for Africa (CDT-Africa), Addis Ababa University, Addis Ababa, Ethiopia

## Correction to: BMC Res Notes (2018) 11:740 10.1186/s13104-018-3844-1

Following publication of the original article [1], the authors reported that one of the authors’ names was spelled incorrectly. In this Correction the incorrect and correct author name are shown. The original publication of this article has been corrected.

Originally the author name was published as:

- Habtom Legese

The correct author name is:

- Haftom Legese
